# Profiling of the microRNA transcriptome in feline whole blood

**DOI:** 10.1038/s41598-025-09478-x

**Published:** 2025-07-31

**Authors:** Åsa Ohlsson, Sofia Hanås, Bodil S. Holst, Julie Lorent, Göran Andersson, Katja Höglund, Anna Tidholm, Ingrid Ljungvall, Jens Häggström

**Affiliations:** 1 Department of Animal Biosciences, Swedish University of Agricultural Sciences, Uppsala, Sweden; 2 Department of Clinical Sciences, Swedish University of Agricultural Sciences, Uppsala, Sweden; 3 Evidensia Specialist Animal Hospital Strömsholm, Strömsholm, Sweden; 4 National Bioinformatics Infrastructure Sweden, Science for Life Laboratory, Department of Biochemistry and Biophysics, Stockholm University, Stockholm, Sweden; 5 Anicura Albano Animal Hospital, Stockholm, Sweden

**Keywords:** miRNAs, Genetics, Biomarkers, Cardiology

## Abstract

Circulating microRNAs (miRNAs) are potential biomarkers for numerous diseases. Characterization of the whole blood (WB) miRNA-transcriptome (miRNome) in cats is lacking, which limits the potential use of miRNAs as biomarkers for diseases such as feline cardiovascular disease. The aims of the present study were to profile and evaluate circulating miRNAs in feline WB by high-throughput sequencing of the total miRNome in WB from twelve domestic mixed breed (DOM) and Norwegian Forest (NFO) cats stringently diagnosed with or without preclinical hypertrophic cardiomyopathy (HCM). A total of 459 mature miRNAs were identified in feline WB, of which 40 were potential novel feline miRNAs. A majority, 85.3%, of the miRNAs showed sequence similarity with human miRNAs. An effect of breed was found, with up to thirteen WB miRNAs being differentially abundant between breeds. The majority of the significant breed-specific miRNAs in feline WB could be associated with regulation of haematopoietic cells. One miRNA, miR-204-5p, was potentially associated with preclinical HCM in NFO cats, but the results need to be confirmed in a larger and sex-unbiased cohort. In conclusion, here we used miRNome-sequencing to identify hundreds of circulating miRNAs in feline WB. Breed should be considered when evaluating the miRNome in feline WB.

**Supplementary Information:**

The online version contains supplementary material available at 10.1038/s41598-025-09478-x.

## Introduction

MicroRNAs (miRNAs) are important regulators of cellular processes. These small non-coding RNAs, approximately 22 nucleotides long, are known to regulate processes such as developmental determination and differentiation, apoptosis, cell cycle and proliferation^1,2^. The mechanism of action of miRNAs are to either target mRNAs for degradation or to inhibit translation by binding to complementary target regions in mRNAs and thereby regulate expression at the post-transcriptional level^3–5^. Abnormal regulation of miRNA expression could lead to cellular malfunction, resulting in a variety of diseases, such as cancer^6^, immune-mediated^6^, neurodegenerative^7^ and heart disease^1,8^. For example, it has been shown that loss of a miRNA pre-processing RNAse III endonuclease, referred to as “Dicer”, can lead to dramatic rearrangement of the human myocardium^1,8^, such as hypertrophy, fibrosis and myofiber disarray^1^. Expression of miRNAs, as well as their target mRNAs, has been shown to be tissue-specific^9–11^ and can be identified in body-fluids such as serum/plasma or urine^11–13^ at concentrations reflecting those observed in the original tissue^12^. Excretion and transport of miRNAs between tissues is facilitated by exosomes^14^ or by specific proteins such as argonaute 2^15^.

Following the discovery that miRNAs are detectable and remain stable in blood^16–18^, several studies have suggested them as promising novel biomarkers in various diseases^19–21^. Hence, identified miRNA-signatures in blood could potentially have a great impact on individualizing future medical treatments^16^. However, it will be difficult to differentiate between background miRNA-signatures in blood and potential circulating biomarkers of interest when basic knowledge of the transcriptome of miRNAs (miRNome) in feline whole blood (WB) is lacking^22^. Currently, no information is available concerning the miRNome in feline WB, which limits the possibility of identifying potentially suitable miRNAs as new biomarkers in cats.

Cardiomyopathy is reported to be the most prevalent cause of heart problems in cats^23,24^, of which hypertrophic cardiomyopathy (HCM) is the most common form^25^. Hypertrophic cardiomyopathy is a heart muscle disease characterized by left ventricular hypertrophy in the absence of other explanations for wall thickening^26^. These changes may result in decreased efficiency of the heart to pump blood, leading to congestive heart failure (CHF), arrhythmia, arterial thromboembolism and/or sudden cardiac death^26^. A significant proportion of cases of HCM in both humans and cats are considered to be familial in origin, where genetic screening for predisposing gene variants may allow identification of individuals at risk^27–30^. However, genetic variation in human miRNAs seed regions have also been shown to influence phenotypes and to be associated with disease^31,32^. Therefore, miRNA may be of importance in disease progression and be of relevance as potential biomarkers in veterinary medicine as well.

The aims of the present study were to profile and evaluate circulating miRNAs in feline WB by high-throughput sequencing of the total miRNome in WB from twelve Norwegian Forest (NFO) and domestic mixed breed (DOM) cats stringently diagnosed with or without HCM.

## Results

In the present study WB-samples from clinically well characterized cats^33,34^ of two breeds (domestic mixed breed (DOM) and Norwegian Forest (NFO) cats) were used to evaluate the WB miRNome. The average age, including standard deviation, of the cats were 8.21 ± 3.70 years and for weight 5.63 ± 1.35 kilos, with no significant differences observed between groups. The samples used for miRNome-study had been collected in PAX-tubes and stored in − 20 °C, according to manufacturer’s instructions, for a median storage time of 0.45 years (range: 0.10–1.68 years) before total RNA extraction and sequencing. As defined in this study, DOM are cats with mostly unknown background, while NFO are pure-breed cats with registered ancestry from a closed breeding-population. The healthy cats used as controls in the miRNome-study were free of heart disease, or any other systemic disease that could cause secondary effects on the heart. The cats classified as affected were in a preclinical state of hypertrophic cardiomyopathy (HCM) and not under medical treatment.

### Identification of miRNAs in feline whole blood

The sequence data was initially evaluated for the need of filtering sequenced reads prior to prediction analyses of miRNAs. This was done by first comparing mapped results from two different aligners; STAR and Bowtie. STAR is a commonly used universal RNA-sequence aligner, while Bowtie is recognized as a short-read aligner (and used by miRDeep2 for miRNA prediction). Both programs provided similar mapping results regardless of reference genome used, and subsequent evaluation of the STAR-mapped reads for assigned features of those mapped sequences indicated no need for additional filtering of data (see Supplementary 1, Fig. S1-2).

Prediction analysis by miRDeep2 identified 60 potentially novel miRNAs in cats, using human as main reference and following a miRDeep2 cut-off score at ≥ 5.0 (see Supplementary 2 for extensive list). Predicted miRNAs that miRDeep2 cannot find a corresponding match for in the main reference will be denoted as novel miRNAs. So, after closer inspection 18 of the 60 novel predicted miRNAs had the same mature sequence as 16 miRNAs identified in mouse and 2 miRNAs in dogs. Also, five of the novel predicted miRNAs have previously been reported in another feline miRNA-study^10^, of which three miRNAs were among the mouse (miR-7064-3p and miR-664-3p) and dog (miR-8891) references used in this study. In total, 40 of the potentially 60 novel miRNAs had previously not been identified in human, dog, mouse or cat. Full lists of identified known and novel miRNAs in feline WB, counts, and a table of the novel miRNAs previously described in another feline study^10^ are presented in Supplementary 2.

### Identification of differentially abundant miRNAs

**Fig. 1:**
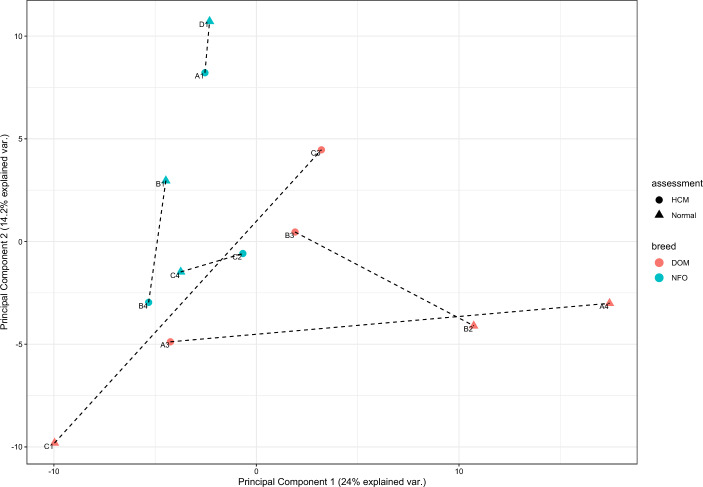
Principal component analysis of the 12 cats included in the miRNome-study. Data is based on normalized counts estimated size factors (to correct for differences in library sizes) of sex-, age- and body weight-matched domestic mixed breed (DOM, orange color) and Norwegian forest (NFO, blue color) cats with (•) and without (▲) preclinical hypertrophic cardiomyopathy (HCM). Matched cats are connected with dashed lines. A row sum cut-off of ≥ 10 counts were used in the analysis.

Out of 459 identified mature miRNAs, 334 remained in the model when miRNAs with a row sum (RS) of < 10 counts were excluded from the analysis. Fifty out of those 334 miRNAs were novel predicted miRNAs (the feline miRNAs B1_2814 and D2_8511 had less than 10 RS counts and were excluded from analysis). Of the miRNAs remaining in the analysis 85.3% miRNA had the same mature sequence as human miRNAs, 11.8% were predicted feline miRNAs, and the remaining 2.9% showed sequence similarity with mouse and dog miRNAs. Stratification analysis revealed no indications of outliers, but indicated a potential breed bias in the dataset, see principal component analysis (PCA)-plot in Fig. 1 (and additional information in Supplementary 1, Fig. S3-4). The different usage of row sum (RS) and row mean (RM) in this study was done to visualise the difference in results based on choice of cut-off. Both methods are used in research.

Initial analyses of data focused on statistical models using single group-variables (~ heart status, ~ breed and ~ sex). These analyses identified no differences in abundance of miRNAs associated with preclinical HCM compared with levels found in WB from healthy cats (HCM *vs.* healthy) for any of the prefiltering cut-offs, but identified up to 13 miRNAs that were differentially abundant depending on breed (NFO *vs.* DOM), see Table 1. Twelve significant miRNAs were identified as differentially abundant between NFO and DOM at a RS cut-off of ≥ 10 (see Table 2), and thirteen at a row mean (RM) cut-off of ≥ 5. Three miRNAs were differently identified between the two cut-off groups; the novel feline A2_1436 observed only in the ≥ 10 RS cut-off group and miR-26b-5p and miR-326 observed only in the ≥ 5 RM cut-off group. Sex differences (male *vs*. female) were only possible to test for in the NFO but was still unbalanced in favour of females (n = 4). Two significant miRNAs were consistently identified as differentially abundant between sex in the NFO, miR-150-5p and miR-146a-5p, with *p* = 0.0028 and *p* = 0.0146 (Benjamini-Hochberg (BH)-adjusted), respectively, for a cut-off ≥ 10 RS counts when data for DOM was excluded.

**Table 1:** Significant over- and underrepresented miRNAs affected by breed and sex in feline WB. Results show significant miRNAs following evaluation with simple single group models and with different prefiltering steps of the data.

*Breed (n* = *12)*					
10 row sum counts			5 row mean counts		
**miRNA**	**log**_2_ **FC**	**padj**	**miRNA**	**log**_2_ **FC**	**padj**
*miR-125b-5p*	4.0^†^	0.039	*miR-125b-5p*	4.0^†^	0.039
*miR-375-3p*	− 1.5^†^	0.039	*miR-375-3p*	− 1.5^†^	0.039
*A2_1436*	− 4.3	0.061	*let-7c-5p*	1.1^†^*	0.091
*miR-144-5p*	1.3^†^*	0.091	*miR-144-5p*	1.3^†^*	0.091
*miR-151a-3p*	− 0.7	0.091	*miR-151a-3p*	− 0.7	0.091
*miR-330-5p*	− 1.0	0.091	*miR-3059-5p*	− 1.8	0.091
*miR-331-3p*	− 0.6	0.091	*miR-330-5p*	− 1.0	0.091
*miR-3613-5p*	1.9^†^*	0.091	*miR-331-3p*	− 0.6	0.091
*miR-99a-5p*	4.5^‡^	0.091	*miR-3613-5p*	1.9^†^*	0.091
*let-7c-5p*	1.1^†^*	0.099	*miR-99a-5p*	4.5^‡^	0.091
*miR-3059-5p*	− 1.8	0.099	*miR-98-5p*	1.0^†^*	0.093
*miR-98-5p*	1.0^†^*	0.099	*miR-26b-5p*	0.6^†^*	0.100
			*miR-326*	− 0.8	0.100

*Sex in NFO (n*** = ***6)*					
10 row sum counts			5 row mean counts		
**miRNA**	**log**_2_ **FC**	**padj**	**miRNA**	**log**_2_ **FC**	**padj**
*miR-150-5p*	1.5^‡^	0.003	*miR-150-5p*	1.5^‡^	0.004
*miR-146a-5p*	1.4	0.015	*miR-146a-5p*	1.4	0.014

A Benjamini-Hochberg (BH) adjusted *p*-value (padj) of ≤ 0.1 was considered significant. Trends validated in additional WB-samples for similar group-comparison are marked with ^†^. Similar trends validated but for other group-comparison (both breeds included for sex-comparison, and only HCM-affected cats compared for breed), are marked with ^‡^. Trends of validation results that are significant are marked with *. FC = fold change; NFO = Norwegian Forest cat.

**Table 2:** Differentially abundant miRNAs according to evaluation with the interaction model.

Interaction model								
*Breed*								
All cats (n = 12)			Healthy cats (n = 6)					
10 row sum counts			10 row sum counts			5 row mean counts		
miRNA	log_2_ FC	padj	miRNA	log_2_ FC	padj	miRNA	log_2_ FC	padj
*let-7f.-5p*	1.8^†^*	0.034	*let-7f.-5p*	1.8	0.034	*miR-151a-3p*	− 1.1	0.023
*miR-151a-3p*	− 1.1	0.034	*miR-151a-3p*	-1.1	0.034	*miR-3613-5p*	3.3	0.023
*miR-3613-5p*	3.3^†^*	0.034	*miR-3613-5p*	3.3	0.034	*let-7f.-5p*	1.8	0.026
*miR-98-5p*	1.7^†^*	0.037	*miR-98-5p*	1.7	0.037	*miR-98-5p*	1.7	0.027
*miR-330-5p*	− 1.4	0.058	*miR-330-5p*	-1.4	0.058	*miR-330-5p*	− 1.4	0.039
*miR-26b-5p*	1.0^†^*	0.079	*miR-26b-5p*	1.0	0.079	*miR-26b-5p*	1.0	0.065
*A2_1436*	− 5.9	0.096	*A2_1436*	− 5.9	0.096			

***Heart status in NFO (n* = *6)***								
5 row mean counts								
**miRNA**	**log**_**2**_** FC**	**padj**						
*miR-204-5p*	− 3.8^†^	0.0624						

Results show significant miRNAs following evaluation with different prefiltering steps of the data. A Benjamini-Hochberg (BH) adjusted *p*-value (padj) of ≤ 0.1 was considered significant. Trends validated in additional WB-samples for similar group-comparison are marked with ^†^, and if statistically significant also marked with *. FC = fold change; NFO = Norwegian Forest cat.

Another statistical model evaluated the combined effect of preclinical HCM and breed (~ breed + heart status). No differentially abundant miRNAs could be identified with that model for either breed or heart status, regardless of prefiltering cut-offs used. The group with the highest number of significant differentially abundant miRNAs with the interaction model (~ breed + heart status + breed:heart status) was again breed, which held true even when only healthy cats were included in the analysis, see Table 2. No significant differentially abundant miRNAs could be identified between HCM-affected and healthy cats in general. One significant miRNA, miR-204-5p, was identified as being differentially abundant in NFO between healthy and HCM-affected cats at prefiltering cut-off of ≥ 5 RM, but not in DOM and not at a cut-off of ≥ 10 RS. Due to the observed weak association between miRNAs in WB and the presence of preclinical HCM; the initial interaction model was tested against the simpler models based on the likelihood ratio test (using a prefiltering of ≥ 10 RS counts). The results showed that the interaction model could potentially be disregarded in favour of a simple model accounting for breed or heart status alone, when the miRNome data was considered.

Several previously reported miRNAs associated with HCM in people were detected in the feline miRNome in WB, such as miR-27a^35^, miR-29a^35^, miR-21^35–37^, and miR-199a-5p^35^, although not identified at significantly different levels of abundance. Full lists of results from the statistical analyses, ordered by BH-adjusted *p*-value, are provided in separate tables in Supplementary 2.

### Target prediction of differentially abundant and novel feline miRNAs in whole blood

The number of human target genes for each miRNA ranged between 3 and 497 with a target prediction score of ≥ 80. The number of feline target genes for each miRNA were between 3 and 470 for the significantly differentially abundant miRNAs. Target genes common between the human- and feline-based lists ranged between none (for miR-144-5p and miR-3613-5p) and 14 (for miR-125b-5p).

The human and feline target gene lists, which included a higher number of target genes, clearly provided stronger support for significant gene ontology (GO) enrichment. The overall most frequently enriched human-based KEGG (Kyoto Encyclopedia of Genes and Genomes^38–40^)-pathway was observed for regulating pluripotency of stem cells, see Table 4. Protein activity and ATP-binding was identified as an important gene target cluster for miR-26b-5p (enrichment score (ES) of 3.03), (Table 3), while Wnt signalling reached the highest significance level for enriched human-based KEGG-pathway for miR-3059-5p (*p* = 0.0016, BH-adjusted). Feline-based gene target analyses did not reach the same level of significance for GO enrichment as in the human-based analyses. The most notable result in terms of significance in the present study was observed for the feline miRNA A2_1436, which had a significant effect on the pathway for cGMP-dependent protein kinase (PKG) signalling (*p* = 0.096, BH-adjusted). For reference, lists of predicted target genes and GO-analyses results for human- and feline-based analyses, as well as lists of target genes for all novel miRNAs in feline WB are presented in separate tables in Supplementary 3.

**Table 3:** The three top gene ontology terms identified to be associated with gene-clusters in DAVID.

miRNA	ES > 2.0	GO-terminology	GO-category
*let-7c-5p*	2.24	cellular response to amino acid stimulus; extracellular matrix structural constituent; endoplasmic reticulum lumen	BP; MF; CC
*miR-26b-5p*	3.03	protein phosphorylation; protein serine/threonine kinase activity; ATP binding	BP; MF; MF
	2.25	Wnt signalling pathway, calcium modulating pathway; posttranscriptional gene silencing by RNA; miRNA mediated inhibition of translation	BP; BP; BP
	2.07	negative regulation of pancreatic juice secretion; positive regulation of ion transmembrane transporter activity; regulation of ion homeostasis	BP; BP; BP
*miR-98-5p*	2.23	cellular response to amino acid stimulus; extracellular matrix structural constituent; endoplasmic reticulum lumen	BP; MF; CC
*miR-204-5p*	3.60	transcriptional activator activity, RNA polymerase II core promoter proximal region sequence-specific binding; positive regulation of transcription from RNA polymerase II promoter; transcription from RNA polymerase II promoter	BP; MF; MF

Data is presented for miRNAs that have clusters with an enrichment score (ES) > 2.0. Results are based on human based gene-target lists. GO = gene ontology; BP = biological process; MF = molecular function; CC = cellular compartment.

### Validation of significant and differentially abundant miRNAs

The significant differentially abundant patterns of miRNA in the feline miRNome in WB was validated by qRT-PCR in a second cohort of 24 cats. All samples of the second cohort were based on EDTA WB, stored between 14 days (0.04 years) up to 16 years in − 80 °C. Three reference miRNAs were used for normalisation, miR-107, miR-423-5p, and miR-30e-5p, which in combination spanned the range of Ct-values for the various quantified miRNAs. The separate assays for the feline-specific miRNA A2_1436 were initially checked by running standard curves of the oligo-control. Based on the results from the quality check one of the assays (Assay 2, see Supplementary 1, Fig. S5) was used for miRNA quantification.

Six of the 24 cats were later excluded from subsequent statistical analyses; one DOM (affected, male) that was affected with non-regenerative anaemia at date of sampling; one NFO (healthy, female) that was shown to have a profound and statistical effect on the sample cohort when storage-time was considered; and four DOM in CHF (see Supplementary 1, Table S4). The cat with anaemia and the cats with CHF were removed due to potential bias imposed on cell-count of various hematopoietic cells in WB due to these clinical presentations. In total eight NFO (equally distributed between healthy and preclinical HCM) and ten DOM (all preclinical HCM) were included in the validation study after statistical analyses had confirmed that storage time had no influence on the included samples (see Supplementary 1, Table S5). These 18 cats had a similar mean age, 7.70 ± 4.77 years, as the cats used in the miRNome-study and no statistical differences was observed within or between the cohort for the validation- and the miRNome-study based on these figures for breed or health groups. In addition, the total sex-ratio was the same between the two studies, with males representing 66.67% of the samples in both cohorts. However, in contrast to the miRNome-study, four of the DOM samples in the validation-study were female, providing a more balanced cohort for the validation- compared to the miRNome-study based on sex, but still biased for males.

The results have been highlighted in Tables 1 and 2 to visualise similar trends and significances for miRNA between cohorts, while detailed information of fold-change and statistical power is provided in Supplementary 1, Table S6. In total, eight miRNAs showed similar trends between the miRNome and the validation cohort when the same types of groups were compared. Five miRNA, miR-144-5p, miR-3613-5p, let-7c-5p, miR-98-5p and miR-26b-5p, showed similar trends of abundance and were also significantly different between breeds, like in the miRNome cohort. Two miRNA, miR-125b-5p and miR-375-3p, showed the same trend but was not statistically significant between breeds, as did miR-150-5p indicate for sex when all cats were evaluated regardless of breed, and miR-99a-5p when breed was evaluated for only HCM-affected cats. Same trend as in the miRNome-study was observed for miR-204-5p (fold change of -0.2), the miRNA associated with preclinical HCM in the NFO, but was not possible to statistically evaluate due to too few samples per group. When comparing the result between the two cohorts, then the results from the interaction model appeared to best describe the observed differences in abundance of miRNA between NFO and DOM with and without HCM.

## Discussion

This is the first study describing the total miRNome of feline WB in cats with or without cardiovascular disease. Four hundred and fifty nine different circulating miRNAs were identified following high-throughput small RNA-sequencing of WB from the twelve sequenced cats, which is a number of miRNAs in line with similar studies of WB in *i.e.* humans^41^ and horse^42^. Forty of the miRNAs identified in WB in cats were considered novel and previously not identified in humans, dogs, mice or cats. Breed had the highest impact on number of significant differentially abundant miRNAs in feline WB, with a maximum of 13 miRNAs shown to have either over- or under-representation in detectable levels between the NFO and DOM cat breeds. Because DOM by far outnumber pedigree cats in the world and are often included in research studies, breed-differences are potentially of importance to consider in future research concerning miRNAs as biomarkers in feline WB.

Breed differences in miRNA-expression patterns have been shown to influence miRNA-profiles in horse blood^43^ and in bovine muscle tissue^44^, but this is the first study to highlight the influence of breed on miRNA-profiles in cats. Feline miRNAs have not been extensively studied, and the limited number of publications have hitherto not addressed potential breed differences^10,13,45–50^.

Both red and white blood-cells express high numbers of miRNA^41,51,52^, where each type of blood-cell has its own specific repertoire of miRNAs^41,51^. The feline WB miRNAs identified in the present study present a mix of miRNAs that are also observed in various types of human blood-cells. For example, miR-326 has been associated with human monocytes, miR-99a-5p with T-cells, miR-151-3p with B-cells, and miR-150 and miR-146a have both been associated with increased levels in B-, T-, and CD56-positive cells^41^. The miRNAs miR-125b, miR-146, miR-330 and miR-26b have been reported in association with reticulocytes^52^, and in addition, the whole let-7 family and miR-144-5p have been reported in association with human erythrocytes^51^. All of these mentioned miRNAs were identified as differentially abundant between cat breeds, and between sex, in the present study, indicating that the majority of the sequenced small RNAs in feline WB are representing miRNAs from various blood-cells. This was further strengthened by the evaluation of enriched gene clusters and genetic pathways by potential targets for these miRNAs (Tables 3 and 4), where the GO-analyses indicate targets of relevance for *e.g.* haematopoietic cells. Further, miR-125b-5p and miR-99a-5p have been associated with inhibition of T-cell activation and promoted T-cell apoptosis^53^, and the let-7 and miR-98-families have been associated with Fas (a death receptor on the surface of cells that leads to programmed cell death) and Fas-mediated apoptosis that is critical for regulation of the immune response^54^. Here it can only be speculated why these blood-cell specific miRNAs differed between breeds, but differences in miRNA profile have been reported in humans with erythrocyte disease, like sickle cell disease^52^, and with different blood-types^55–58^. There are known blood-type differences^59,60^ and occurrence of gene variants associated with blood-cell related diseases, like pyruvate kinase deficiency^61^, in cats and these may have had an impact on the miRNA profile in this study. For example, in humans miR-331-3p has been shown to be important for regulation of ABO blood-type^57^, and miR-98 for RH-factor^58^, two miRNA that were shown to be significantly different between cat breeds in this study. Blood-type status or presence of the pyruvate kinase deficiency variant among the cats included in this study was unknown at the time of sampling. Further research is needed to elucidate why blood-cell specific miRNAs differs between cat breeds.

**Table 4:** Significantly enriched KEGG-pathways associated with genes targeted by the stated miRNAs.

miRNA	KEGG-pathway	BH-adjusted *p*-value ≤ 0.1
*let-7c-5p*	Signalling pathways regulating pluripotency of stem cells	0.053
	PI3K-Akt signalling pathway	0.060
*miR-98-5p*	Signalling pathways regulating pluripotency of stem cells	0.056
	PI3K-Akt signalling pathway	0.065
*miR-330-5p*	Rap1 signalling pathway	0.044
	Ras signalling pathway	0.044
	Regulation of actin cytoskeleton	0.082
*miR-3059-5p*	Wnt signalling pathway	0.0016
	Dopaminergic synapse	0.065
	Pathways in cancer	0.065

Pathways with a BH-adjusted *p*-value of ≤ 0.1 are presented. Extended information is presented in miRNA-specific tables in Supplementary 3.

To verify the results of the miRNome-study, WB from additional NFO and DOM were evaluated with qRT-PCR. Similar trends could be observed for up to 9 of the total 17 miRNAs that were re-evaluated. Five of these miRNAs are among the ones associated with blood-cells mentioned above, identified to differ between breeds. In extension, four of these (miR-144-5p, let-7c-5p, miR-98-5p and miR-26b-5p) also showed significant power associated with these observed trends, strongly backing up the results from the miRNome-study. Preferably the validation samples should have been of the same collection type and stored for similar time-frame, however such samples were not available. Still, despite using EDTA-WB for the validation, where samples had been stored up to 16 years at − 80 °C conditions, similar trends could be established between the two cohorts for more than half of the miRNA. If only miRNA identified based on the more complex interaction model was considered, then trends for 62.5% of the miRNA could be confirmed. Interestingly, no significant differences could be established for the EDTA-sampled cats based on time of storage, except for one cat only stored for 14 days in − 80 °C. Reports evaluating long-time storage stability are limited, but it has been shown that stability of miRNA for a storage-period of nine months has been successful for serum and plasma samples, while WB-samples may vary^17^. Time therefore pose a potential problem to miRNA-stability in the long-time stored WB, even if that did not appear to be prominent for the evaluated miRNA in the present study. Evaluating the effects of long-time storage of EDTA-WB should therefore be addressed in future research. Another influencing factor on levels of miRNA in the EDTA-WB could be undiscovered comorbidities, not expected to affect the heart status, but which could impact the blood-cell variables (e.g. like dental/gingival problems). On a final note, in neither the miRNome- or the validation cohort was the exact pedigree background of the DOM known. It could be that several of these cats in both cohorts had NFO background, which obviously could skew the results.

Several variables, besides breed, are obviously of importance when identifying miRNA-profiles. Examples of such variables include the intra-individual factors age^62,63^, body-weight^62,64^, and sex^62,63^, as well as type of organ investigated^10,65^, disease status^66^ and pre-analytical variables like haemolysis in a serum/plasma sample^22^. In this study paired samples of healthy and preclinical HCM-cats were used for the miRNome-study. This paired-sample approach was chosen to limit the potential impact of other variables of importance on miRNA-profiles when HCM-status was also of interest to investigate. For HCM, several factors are known to influence disease development in cats, such as sex^26^, breed-specific genetic variants^26,28,29^ and age^26^. In the miRNome-study, sex was not evenly distributed and therefore introduced a potential bias in the comparison between breeds, especially in the comparison between only healthy cats in the interaction model. However, none of the identified miRNAs significantly associated with sex in the NFO were among the miRNAs identified between breeds. Also, the validation study later revealed contradicting trends for these miRNAs in NFO in terms of sex, potentially indicating that sex may not have had a large impact on the overall comparison between breeds in the miRNome-study. However, it was observed that miR-150-5p was significantly affected by sex among DOM in the validation study, where all of the evaluated cats were also diagnosed with HCM. Further studies are therefore needed to evaluate the potential effect that sex may have on miRNA expression between and within breeds, with or without HCM.

In a previous study evaluating miRNAs using a human-based array, several miRNAs in feline serum were shown to be differentially expressed between healthy cats and cats with stable congestive heart failure caused by HCM^50^. No DOM were included in that study and NFO were unevenly distributed between healthy and HCM-affected cats: 7/12 healthy controls were NFO, whereas no NFO were included among the cats with HCM. The eleven differentially expressed miRNAs in that study might have been influenced by an effect of breed as well as HCM, but none of them were among the significant miRNAs observed in the present miRNome-study. However, in the present study, one miRNA; miR-204-5p, was identified as significantly differentially abundant in the interaction model when healthy and preclinical NFO cats were compared, but not when heart status was compared regardless of breed. The trend for miR-204-5p was similar in the validation study, but the low number of samples prevented statistical evaluation. Interestingly, another miRNome study addressing the expression of miRNA in feline heart tissue also identified miR-204-5p as differentially abundant between healthy and HCM-affected cats^47^. The trend of decreased levels of miR-204-5p in heart tissue of affected cats is in agreement with what was observed in feline WB in this study. This miRNA, miR-204-5p, is interesting because it has previously been associated with a risk of developing heart disease^67^. For example, it has been shown to negatively affect apelin-receptor signalling regulation^67^, which could lead to cardiac dysfunction and hypertrophy, and be involved in regulation of exercise-induced hypertrophy of the heart in rats^68^. An explanation for the overall weak miRNA-signature between healthy and HCM-affected cats that was observed in this study could be that the cats with HCM in this miRNome-study were in the preclinical phase of the disease, hence not releasing sufficient amounts of miRNAs into the circulation, preventing conclusive association with remodelled or damaged heart tissue into the blood stream to be detected. The miRNA-signature is likely to be enhanced as disease progresses^66^, which may account for the observed differences in the result of the present study compared to the studies mentioned above; where the cats had more severe signs of HCM disease^47,50^. In our validation study, only four cats had more severe HCM, presenting with CHF signs. Unfortunately, these cats were DOM and healthy DOM was not available for comparison of the level of miR-204-5p. It would be interesting to evaluate the levels of miR-204-5p in a larger cohort of healthy DOM for comparison, to see if the results in feline WB of HCM-affected cats can mirror the results from the heart miRNome study^47^. Some additional, previously reported, miRNAs suggested as biomarkers for HCM in human blood, like miR-27a^35^, miR-29a^35^, miR-21^35–37^, and miR-199a-5p^35^, were detected in the feline WB miRNome, but their abundance was not found to be different in HCM-affected cats compared to healthy cats. Another reason for the weak miRNA-association between the cats with HCM and the healthy cats in the present study could be that too few cats were included in a study heavily influenced by blood-cell specific miRNAs. For identification of potential biomarkers associated with preclinical HCM, the use of serum and/or plasma is likely more appropriate than WB.

In the present study we made use of the fact that miRNAs are conserved and show high sequence similarity between species^16,65,69,70^. Therefore, human, mouse and dog miRNAs were used as references when screening for miRNAs in feline WB in the present study. This sequence similarity was reflected in the results, where 85.3% of the identified miRNAs in feline WB (cut-off ≥ 10 row sum counts) shared the same mature sequence with human miRNAs. Only 11.8% (40 out of 339) of the miRNAs in feline WB could not be mapped to any of the references used, thereby potentially representing 40 miRNAs that could be unique to cats. This indicates that veterinary medicine likely could benefit from human miRNA-arrays in identifying a large proportion of feline miRNA. Currently only one study^50^ has previously utilized human microarrays and applied it for miRNA screening in cat serum of healthy and HCM-affected individuals. However, there is a risk that such arrays fail to identify feline-specific miRNAs associated with disease. Further studies comparing the results from the human arrays with matched miRNome-sequenced samples could therefore elucidate this assumption further.

The feline reference genome is not as well annotated as the human reference genome, limiting the possibilities to identify potential 3’-UTR targets in the feline genome. The mammalian 3’-UTRs are often conserved^69,71,72^. Hence, in addition to target prediction analysis of feline-specific miRNAs against the feline 3’-UTR-sequences, the present study also evaluated the potential mRNA targets of the identified miRNAs against human targets. This was performed to broaden the understanding of the potential impact of these identified miRNAs also in cats, as discussed above in relation to blood-specific miRNAs. The results from the GO-analysis provided extensive information about various significant gene clusters, pathways and gene function for the predicted miRNA-targeted mRNAs in humans, but was less clear for the feline-based analyses. Most of the identified biological features from the GO-analyses appear to be related to hematopoietic cells, rather than miRNAs transported in *e.g.* exosomes related to preclinical HCM, even if HCM-related miRNAs could be detected. The feline-specific miRNA A2_1436 was differentially abundant depending on breed in the miRNome-study and was significantly associated with the genetic pathway for cGMP-PKG signalling. This pathway has been shown to be important for *e.g.*smooth muscle relaxation^73^ and platelet function^74^, but the significance and trend could not be validated in the second cohort. Further research is needed to clarify the role of A2_1346 in preclinical and clinical HCM and its relevance in feline WB.

The main limitations of this study include a small sample size, inclusion of two cat groups (DOM and NFO) and different sex representation in each group. All these facts together have limited the statistical analyses and the statistical power to show significant results. Blood was only collected once, making evaluation of biological variation in miRNA levels in each individual cat in WB not feasible. Also, some of the discrepancies in results between the two cohorts of WB in this study may be attributed to differences in sample tubes and storage, as well as bioinformatical filtering of isomiRs in miRNome-samples *vs*. very sequence-specific quantification of miRNA of the samples in the second cohort.

In conclusion, circulating previously known and novel miRNAs in feline WB were identified by miRNome-sequencing. Breed was associated with circulating miRNAs in cats. The miRNA miR-204-5p could only be associated with preclinical HCM in NFO, not in DOM. The indicated targets for many of the identified miRNAs appear to be mRNAs expressed in haematopoietic cells. The reported miRNAs in this study serve as an initial reference for future studies aimed at identifying feline-specific miRNAs as biomarkers of disease.

## Materials and methods

The study was approved by the Uppsala Animal Experiment Ethics Board Sweden (No. C267/5, C103/10, C2/12, C137/13, C12/15 and Dnr 5.8.18–04,682/2020) and all cats have been handled and evaluated according to ARRIVE guidelines. Informed written consent authorizing participation of privately-owned cats in the study was obtained from the owner of each cat. The cats included in the miRNome-study are part of a population that has previously been extensively described^33,34^. The cats were treated as blinded samples during sequencing and bioinformatical analyses, unless characteristics were needed for a given evaluation. All experiments and laboratory procedures at the Swedish University of Agricultural Sciences were performed in accordance with guidelines and regulations defined by the Swedish Research council (www.vr.se).

### Study population and design

Six neutered healthy cats were matched with six preclinical HCM-affected cats based on breed, sex, age, and body-weight, see Table 5. These matched variables were considered important to account for in the experimental design, because similar factors have been shown to impact differentially expressed miRNAs in people^62,63,75^. To address this analysis was later performed on the miRNome-data to identify potential stratifications, visualized in a PCA-plot (see Fig. 1), also, statistical analysis was performed to rule out significant differences between breed and heart status based on age and body-weight of the groups. For detailed information about all cats used in the miRNome-study see Table S1 in Supplementary 1. These cats underwent blood-pressure measurement, physical examination, and echocardiography prior to blood-sampling. Haematology and biochemistry evaluation was performed on samples from all cats^33^. The diagnosis of preclinical HCM was based on previously published echocardiographic criteria^26^ in the absence of systolic arterial hypertension (defined as blood pressure > 160 mmHg) or abnormal haematology or blood biochemistry (i.e. increased serum concentrations of thyroxine). Cats were excluded if clinical signs of decompensated CHF, thromboembolism, congenital cardiac disease, other acquired cardiovascular disorders, equivocal findings concerning the presence of hypertrophy of the left ventricle, severe dental disease or clinically relevant organ-related or systemic diseases were present. Cats receiving medical therapy were not included in the study. To clarify, no cats with clinical signs of CHF were included in the miRNome-study.

**Table 5:** Signalment characteristics of cats included in the miRNome-study.

Cat ID	Assessment	Breed	Age (year)	Sex	Weight (kg)
A1	HCM	NFO	6.45	Female	6.0
A3	HCM	DOM	8.96	Male	6.1
A4	Normal	DOM	10.7	Male	5.2
B1	Normal	NFO	8.87	Female	4.7
B2	Normal	DOM	5.22	Male	7.3
B3	HCM	DOM	4.32	Male	5.8
B4	HCM	NFO	12.0	Female	4.6
C1	Normal	DOM	13.8	Male	4.5
C2	HCM	NFO	2.43	Male	8.4
C3	HCM	DOM	13.7	Male	4.4
C4	Normal	NFO	3.73	Male	7.0
D1	Normal	NFO	8.30	Female	3.6

Extended information is presented in Supplementary 1, Table S1. DOM = domestic mixed breed cat; NFO = Norwegian Forest cat.

### Collection of whole blood

Whole blood was collected in PAXgene blood RNA System (Qiagen Inc., Germantown, MD, USA) tubes by venipuncture of the cephalic vein in non-sedated, minimally restrained cats. The tubes were gently inverted 8–10 times immediately after blood collection. Each tube containing 1.5–2.0 mL of WB and 6.9 mL of PAXgene Blood RNA preservative solution additives. The tubes were frozen and stored in − 20 °C until date of RNA-extraction for a median storage time of 0.45 years (range 0.10–1.68 years).

### Total RNA isolation from whole blood and sequencing library preparation

Total RNA was extracted from the PAXgene-tubes following the manual protocol for PAXgene Blood miRNA kit (PreAnalytiX, Qiagen, Hilden, Germany). Speed of centrifuge was 13,000 × G throughout the protocol. Extracted total RNA-samples were stored in − 80 °C until library preparation was initiated (one month). TapeStation 2200 and RNA ScreenTape® (Agilent Technologies, Santa Clara, CA, USA) were used for quantification and quality assessment of the RNA-samples prior to library preparation. Samples with a RIN-value of 7.7 or higher were included in the miRNome-study.

Libraries were prepared from 0.2 μg of total RNA using the NEXTflex™ Small RNA-Seq Kit v3 (Bioo Scientific Corporation, Austin, TX, USA) according to manufacturer’s instructions, generating stranded sequences for sequencing. Quantifications of libraries were performed with Quant-iT™ PicoGreen™ dsDNA Assay Kit (Invitrogen, Paisley, UK) and evaluated on Wallac Victor^2^ 1420 microplate reader (PerkinElmer, Waltham, MA, USA) at the appropriate wavelength. Libraries were normalized to 2 nM and pooled prior to sequencing. Paired-end sequencing data was generated on a mid-output flow-cell, run on Illumina NextSeq550 (Illumina Inc, San Diego, CA, USA).

### Bioinformatic data processing and count-generation of known and novel miRNAs

**Fig. 2:**
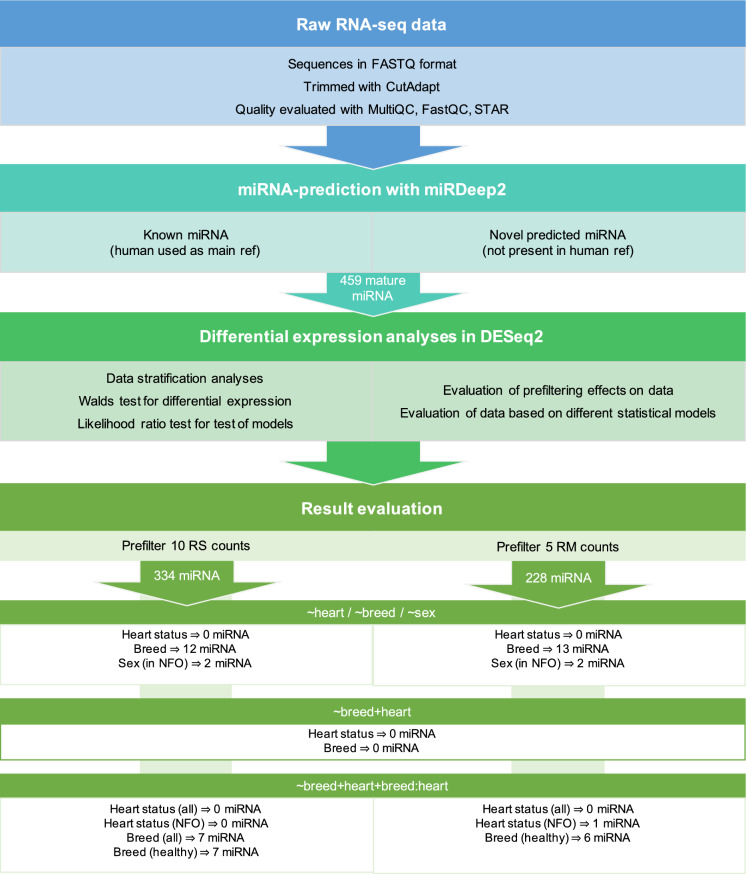
Schematic overview of bioinformatical evaluation of the feline whole blood miRNome. Indicated in the figure are the number of miRNAs represented in a given step. RS = row sum, RM = row mean, NFO = Norwegian Forest cat, and all = all cats, regardless of breed.

The generated bcl-files from the sequencing were converted to fastq-files and de-multiplexed using bcl2fastq 2.20.0 with the option “-no-lane-splitting”. Sequencing adaptors were trimmed with Cutadapt 3.3^76^, according to the library-kit manufacturer’s recommendations. In short, forward and reverse adaptors were removed, followed by trimming of the four randomly added nucleotides to the sequences. Reads shorter than 17 nucleotides were excluded from further processing. FastQC 0.11.9 and multiqc 1.9^77^ was used for quality check of the sequence data. The pipeline used for data handling in this study is schematically presented in Fig. 2.

Identification and quantification of known and novel miRNAs was performed with miRDeep2^78^ 2.0.1.2 utilizing *Felis catus* 8.0 (GCA_000181335.3) as reference genome. The accuracy of the reference genome sequence is important for novel discovery of miRNA. To verify that reads mapped to miRNA, regardless of reference genome in use, and to assess if the miRNome-data needed additional filtering prior to subsequent prediction analyses, trimmed reads were also mapped to the *Felis catus* 9.0 (GCA_000181335.4) reference genome using gtf-file (release 103) from Ensembl and STAR 2.7.2b^78^ as the aligner. The STAR-mapped reads were assigned to gene_biotype using featureCounts^80^. Mapped reads were illustrated with plots. For more detailed information see Supplementary 1.

Mature and hairpin sequences were downloaded from miRBase^81,82^ (release 22.1), with human (*Homo sapiens*) denoted as main reference, and mouse (*Mus musculus*) and dog (*Canis familiaris*) denoted as close relative references according to miRDeep2 definition. Main reference miRNAs identified in the dataset were classified as predicted known miRNAs, and miRNAs previously not described in the main reference were classified as novel miRNAs by miRDeep2. Mature and precursor pairs of miRNAs with multiple rows were excluded from the analysis (two human miRNAs). For each mature miRNA, when multiple quantifications were provided from different precursors, the one with the least total count was kept in the data. Only novel miRNAs with a miRDeep2 score of ≥ 5.0 was included in the count-file generated for subsequent differential expression analyses. For more information on parameters used see Supplementary 1.

### Identification of differentially expressed miRNAs

DESeq2 1.36.0^83^ was used to identify differentially abundant miRNAs in the miRNome-data from feline WB. Prior to normalization and differential expression analysis, data was pre-filtered for levels of row sums, *i.e. *the total sum of counts for one miRNA for all samples. DESeq2 perform independent filtering while estimating dispersions, so pre-filtering is not needed but can speed up the analysis according to the manual and provide an initial count cut-off level for miRNAs to be kept for the statistical analysis. The miRNA-counts were normalized using estimated size factors for evaluation of sample stratifications and to perform differential expression analysis based on the Wald test, a hypothesis test used in DESeq2 to compare two groups. Different statistical models were applied to the data, including simple group-models for heart status (HCM *vs.* healthy), breed (NFO *vs.* DOM) and sex (Male *vs.* Female), and a model accounting for breed and heart status including an interaction factor. Sex was not included in the interaction model due to uneven distribution between breed groups. The interaction model, based on breed and heart status, was compared with the simpler models for these groups by performing a likelihood ratio test included in DESeq2. The subsequent models used in the data analyses only included breed or heart status. As per default in DESeq2, the *p*-values were corrected for multiple testing using the BH-method with a false discovery rate (FDR) of ≤ 0.1. Results from the DESeq2-analyses were listed in tables or as graphical illustrations.

### Target prediction of significant differentially expressed miRNAs and identified novel miRNAs

The 3’-UTR of mRNA is a poorly annotated feature in cats compared to humans. Therefore, miRDB^84,85^ version 6.0 was used to search for predicted targets of the significant differentially abundant human miRNAs identified in feline WB through the miRDBs web server interface. mRNAs with a target prediction score of ≥ 80, according to suggested recommendations, were saved for further gene ontology (GO) analysis. Also, novel and significantly known miRNAs were evaluated for feline specific 3’-UTR targets with IntaRNA 3.2.1^86^, with the seed constrain option “–seedBP” set to 7, and not allowing bulges “–seedNoGU”. 3’-UTR sequences from the feline reference genome (GCA_000181335.4) were downloaded from Ensembl and used as screening-targets in IntaRNA. Only 3’UTR-targets estimated to have a perfect match for the seed-region, high probability of accessibility to the target sequence, and a miRNA-interaction with high stability (*e.g.* low energy) were kept. A probability score of ≤ 0.05 was used as cut-off for accessibility to the target seed sequence and a tight energy constrain^86^ (cut-off at − 4.8 kcal/mol, estimated for a seed of 7 bp) was used as cut-off for miRNA-target interaction. The mature significant differentially expressed human miRNA-sequences were evaluated with IntaRNA, to evaluate if similar genes could be identified between methods. The human- and feline-based lists of predicted miRNA target genes were analysed for GO enrichment in DAVID 6.8^87,88^ as gene lists without providing a miRNA background list. The main focus of the result was cluster analysis. Pathway analysis (KEGG), basic evaluation of biological processes (BP), cellular compartment (CC) and molecular function (MF) for each list of target genes were investigated as well. For all novel miRNAs, predicted target-gene lists were generated with similar seed constrain as above, but with default settings for other parameters. To identify GO, the EASE Score (a modified Fisher Exact Test) was used at *p* ≤ 0.05 in DAVID.

### Validation of results from miRNome-sequencing

The differentially and significantly abundant patterns of miRNA in the feline miRNome was validated by qRT-PCR. Nine NFO and fifteen DOM cats were initially included in the validation, based on WB from EDTA-samples long-time stored in − 80 °C. Five of the NFO cats were assessed as normal, of which three were male and two were female. The other four NFO were assessed with preclinical HCM and were equally distributed between sex. The samples were stored between two weeks (0.04 years) and up to 16 years in − 80 °C. All DOM were assessed with HCM (five females and nine males), of which four were in CHF (two males and two females) and the other cats diagnosed as being preclinical. Storage time in − 80 °C for DOM-samples was between 5.0 and 16 years. Information about the cats used in the validation is provided in Table S2, Supplementary 1,

In brief, total RNA was obtained from 100 µl EDTA-WB for each sample, following optimisation of sample volume, according to manufacturer’s instructions (miRNeasy Mini kit, Qiagen). Extracted samples were stored in − 80 °C until preparation of cDNA, which was performed according to instructions for the miRCURY LNA RT-kit (Qiagen). Input level of total RNA was optimised to 5 ng/reaction and amplification protocol was run according to manual. Generated cDNA-samples were stored in − 20 °C. For qRT-PCR, both panels and stand-alone assays were developed and obtained from Qiagen based on the miRCURY LNA miRNA SYBR Green PCR-kit. Evaluation of spike-in controls, in-plate controls and five cat-specific controls (miR-107, miR-191-5p, miR-26a-5p, miR-423-5p, miR-16-5p, miR-30e-5p, not significantly different in expression between samples and spanning between high and low count levels) were picked from the miRNome count-data and included in all plates. Master mixes and dilutions of samples were made in agreement with manufacturers recommendations and cycling run according to provided temperatures and time-frames. Two separate cat-specific assays for A2_1432 were developed and obtained from Qiagen and an oligo-control for A2_1432 was ordered from TAG Copenhagen (TAG Copenhagen, Frederiksberg, Denmark). The oligo-control was converted to cDNA in agreement with protocol used for the WB-samples.

Normalisation of Ct-values was performed according to recommendations from Riedel et al.^89^ for use of multiple reference genes. Normalized values were statistically evaluated in JMP (JMP Statistical Discovery LLC, Cary, NC, USA). Wilcoxon/Kruskal–Wallis test was used for comparing sample groups, following initial stratification analyses of the dataset based on a step-wise regression model. Samples indicated to be affected by storage time, anaemia or more severe form of HCM than pre-clinical stage were excluded.

## Supplementary Information

Supplementary Material 1.

Supplementary Material 2.

Supplementary Material 3.

## Data Availability

RNA-seq data have been deposited in the ArrayExpress database at EMBL-EBI under the accession number E-MTAB-12138 (https://www.ebi.ac.uk/arrayexpress/experiments/E-MTAB-12138/). Please contact the corresponding author if assistance is needed to access data from the study.
