# Supplementary material for: Profiling of the microRNA transcriptome in feline whole blood
Source: Sci Rep. 2025 Jul 31;15:27962. doi: 10.1038/s41598-025-09478-x (PMC12313912; doi:10.1038/s41598-025-09478-x)
Supplement: Supplementary file 1 — Supplementary Material 1. [file 41598_2025_9478_MOESM1_ESM.docx]

**Profiling of the microRNA transcriptome in feline whole blood**

*Åsa Ohlsson, Sofia Hanås, Bodil S. Holst, Julie Lorent, Göran Andersson, Katja Höglund, Anna Tidholm, Ingrid Ljungvall, Jens Häggström*

**Supplementary 1**

**Information about cats included in the study**

**Table S1. Individual feline characteristics and echocardiographic variables in the twelve cats used for miRNome sequencing**. Abbreviations; HCM=hypertrophic cardiomyopathy (pre-clinical); DOM=domestic mixed breed cat; NFO=Norwegian Forest cat; NF=neutered female; NM=neutered male; SH=shorthair; LH=longhair; ID=identification of the individual cat; BCS=body condition score; I=living indoor only; O=allowed access to outdoors; HR=heart rate auscultation; SBP=systolic blood pressure; LA/Ao=left atrial-to-aortic root diameter ratio ; IVSd=interventricular septum in diastole; IVSd_inc%_=percentage increase interventricular septum in diastole ; LVIDd=left ventricular internal diameter in diastole ; LVIDd_inc%_=percentage increase left ventricular internal diameter in diastole ; LVFWd=left ventricular free wall in diastole ; LVFWd_inc%_=percentage increase left ventricular free wall in diastole ; FS=fractional shortening; Septum 2D=two-dimensional echocardiographic measurement of septum; SAM=systolic anterior motion of the mitral valve.

| **ID** | **A3** | **B3** | **C3** | **A1** | **B4** | **C2** | **A4** | **B2** | **C1** | **B1** | **C4** | **D1** |
| --- | --- | --- | --- | --- | --- | --- | --- | --- | --- | --- | --- | --- |
| Breed | DOM | DOM | DOM | NFO | NFO | NFO | DOM | DOM | DOM | NFO | NFO | NFO |
| Sex (NF/NM) | NM | NM | NM | NF | NF | NM | NM | NM | NM | NF | NM | NF |
| SH/LH | LH | SH | SH | LH | LH | LH | SH | SH | SH | LH | LH | LH |
| BCS (1-9) | 7 | 7 | 5 | 7 | 6 | 7 | 5 | 7 | 5 | 6 | 6 | 5 |
| I/O | 1/0 | 0/1 | 0/1 | 0/1 | 1/0 | 0/1 | 0/1 | 0/1 | 0/1 | 1/0 | 1/0 | 0/1 |
| Age (years) | 9.0 | 4.3 | 13.7 | 6.5 | 12 | 2.4 | 10.7 | 5.2 | 13.8 | 8.9 | 3.7 | 8.3 |
| Weight (kg) | 6.1 | 5.8 | 4.4 | 6.0 | 4.6 | 8.4 | 5.2 | 7.3 | 4.5 | 4.7 | 7.0 | 3.6 |
| Heart status | HCM | HCM | HCM | HCM | HCM | HCM | Normal | Normal | Normal | Normal | Normal | Normal |
| HR (bpm) | 200 | 200 | 140 | 180 | 160 | 112 | 120 | 128 | 136 | 180 | 136 | 140 |
| SBP (mmHg) | 130 | 131 | 148 | 130 | 137 | 107 | 130 | 124 | 151 | 140 | 136 | 139 |
| Murmur (yes/no) | Yes | Yes | Yes | Yes | Yes | Yes | No | No | No | No | No | No |
| LA/Ao | 1.5 | 1.0 | 1.0 | 1.1 | 1.1 | 1.2 | 1.1 | 0.9 | 1.2 | 1.0 | 1.1 | 1.3 |
| IVSd (mm) | 8.9 | 6.4 | 6.3 | 6.5 | 5.8 | 9.1 | 3.9 | 4.7 | 4.0 | 3.3 | 4.3 | 4.0 |
| IVSd _inc_ (%) | 117.7 | 58.7 | 64.5 | 59.6 | 51.2 | 109 | -1.3 | 9.8 | 3.7 | -15.5 | 2.9 | 8.6 |
| LVIDd (mm) | 14.7 | 15.9 | 12 | 10.5 | 10.6 | 15.6 | 14.1 | 19.9 | 16.8 | 14.7 | 19.6 | 15.1 |
| LVIDd _inc_ (%) | -14.5 | -6.2 | -23.9 | -38.6 | -33.6 | -16.5 | -14.4 | 10.5 | 5.9 | -8.4 | 10.0 | 0.9 |
| LVPWd (mm) | 6.9 | 7.3 | 7.4 | 6.4 | 4.8 | 9.4 | 3.9 | 4.7 | 4.0 | 3.3 | 4.2 | 3.4 |
| LVPWd _inc_ (%) | 68.5 | 81 | 97.1 | 56.9 | 25.5 | 112.0 | -0.6 | 9.1 | 4.1 | -14.5 | -1.6 | -5.4 |
| FS (%) | 42 | 47 | 72 | 65 | 54 | 62 | 50 | 47 | 55 | 51 | 50 | 63 |
| Septum 2D (mm) | 9.8 | 8.0 | 8.9 | 6.7 | 6.5 | 9.1 | 4.2 | 4.4 | 4.3 | 3.3 | 4.9 | 3.4 |
| SAM (yes/no) | No | Yes | Yes | Yes | Yes | Yes | No | No | No | No | No | No |
| Storage (years) | 0.15 | 0.64 | 0.54 | 0.11 | 0.42 | 0.42 | 0.90 | 1.68 | 0.56 | 0.19 | 0.48 | 0.27 |

**Table S2. Individual characteristics for the cats used in the validation study**. Abbreviations; HCM=hypertrophic cardiomyopathy (pre-clinical, unless stated otherwise); CHF=congestive heart failure; DOM=domestic mixed breed cat; NFO=Norwegian Forest cat; FORL= feline odontoclastic resorptive lesion.

| **ID** | **Breed** | **Sex** | **Age (years)** | **Heart status** | **Storage time (years)** | **Comments** |
| --- | --- | --- | --- | --- | --- | --- |
| NFO1 | NFO | F | 1.4 | Normal | 0.04 | ***EXCLUDED*** |
| NFO2 | NFO | F | 3.6 | Normal | 3 |  |
| NFO3 | NFO | M | 15.4 | Normal | 12 |  |
| NFO4 | NFO | M | 6.0 | Normal | 0.5 |  |
| NFO5 | NFO | M | 11.2 | Normal | 12 |  |
| NFO6 | NFO | F | 5.1 | HCM | 15 |  |
| NFO7 | NFO | M | 3.6 | HCM | 12 |  |
| NFO8 | NFO | F | 8.1 | HCM | 12 |  |
| NFO9 | NFO | M | 12.3 | HCM | 5 |  |
| DOM1 | DOM | F | 14.1 | HCM | 14 | Dental fracture, FORL, arthrosis |
| DOM2 | DOM | M | 9.3 | HCM | 5 | Periodontitis, FORL |
| DOM3 | DOM | F | 0.7 | HCM | 8 |  |
| DOM4 | DOM | F | 0.4 | HCM+CHF | 8 | Cat in CHF - ***EXCLUDED*** |
| DOM5 | DOM | M | 1.4 | HCM | 10 | Chronic urinary problems |
| DOM6 | DOM | M | 1.5 | HCM | 16 | Fracture of femur |
| DOM7 | DOM | M | 9.9 | HCM+CHF | 14 | Cat in CHF - ***EXCLUDED*** |
| DOM8 | DOM | M | 13.1 | HCM+CHF | 8 | Cat in CHF - ***EXCLUDED*** |
| DOM9 | DOM | M | 10.9 | HCM | 12 |  |
| DOM10 | DOM | F | 0.9 | HCM | 12 |  |
| DOM11 | DOM | M | 9.9 | HCM | 13 |  |
| DOM12 | DOM | M | 12.3 | HCM | 12 |  |
| DOM13 | DOM | F | 1.6 | HCM+CHF | 11 | Cat in CHF - ***EXCLUDED*** |
| DOM14 | DOM | M | 12.1 | HCM | 12 |  |
| DOM15 | DOM | M | 6.2 | HCM | 8 | Nonregenerative anemia - ***EXCLUDED*** |

**Bioinformatic evaluation extended information**

*Quality control of sequencing reads*

Small RNAs shorter than 17 nucleotides were removed from the sequencing data while trimming adapters from reads. The percentage of reads that were too short was similar for all samples and was on average 1.5%. To verify that most reads map to miRNAs (rather than *e.g.* rRNA), trimmed reads were aligned to the feline genome using STAR and assigned to gene_biotype using featureCounts. The STAR alignment was performed using the following parameters (in order to prevent exclusion of short reads) “–outFilterMismatchNoverLmax 0.05 –outFilterMatchNmin 16 –outFilterScoreMinOverLread 0 –outFilterMatchNminOverLread 0 –alignIntronMax 1 –outSAMtype BAM SortedByCoordinate”. The STAR index was built from Felis catus 9.0 fasta and gtf file (Felis_catus.Felis_catus_9.0.dna.toplevel.fa and Felis_catus.Felis_catus_9.0.103.gtf) downloaded from Ensembl release 103. Mapped reads from STAR-alignment are presented in *Fig. S1*.

The miRDeep2 mapper.pl script was used for preprocessing and genome mapping of sequencing reads with options -j (remove reads containing anything other than a,c,g,t,u,n,A,C,G,T,U,N) and -q (map with one mismatch allowed in the seed region). Felis catus 8.0 was used as reference genome. All other parameters were kept as default values. Mapped reads from concatenated data, aligned to the reference genome with Bowtie, is presented in *Fig. S2*.

**Figure S1. Visualization of mapped reads with STAR and Bowtie**. Same dataset is mapped with two different aligners, I) STAR (utilizing Felis catus 9.0 as a reference) and II) Bowtie (built in aligner in miRDeep2, utilizing Felis catus 8.0 as a reference). Mapped, unmapped and reads filtered out are indicated in the legends. Samples are coded according to *Table S1* above.

To assign features to the mapped reads featureCounts was used with the options “-t exon -g gene_biotype -s 0 -M”. Not all reads could be assigned a biotype, but of those that could the majority of reads were assigned to miRNA. Plots of assigned features and the reads aligning with miRNA are presented in *Fig. S2*.

**Figure S2. Visualization of assigned features to the mapped reads with STAR.** I) Plot of assigned features. II) Plot of assigned biological feature for those that could be assigned. Samples are coded according to *Table 1*.

*Identification of known and prediction of novel miRNA*

Mature and hairpin-sequences to be used in the analysis’s were downloaded from miRBase release 22.1. Human miRNAs were considered as known miRNAs and mouse and dog are denoted as related species, according to miRDeep2-definition. miRDeep2 requires small processing of fasta files (genome and miRNA files) to remove white spaces in identifiers and remove trailing spaces at the end of files. The miRDeep2 scripts remove_white_space_in_id.pl and fastaparse.pl were used for these purposes, respectively. The mirdeep2.pl script was used for the identification of novel miRNA step with option -c (disable randfold analysis). Preprocessing and mapping to genome (mapper.pl) with miRDeep2 was performed individually for each sample using the same settings as described above for concatenated reads. Known (human) and novel miRNAs were quantified using miRDeep2 quantifier.pl script with option -k (considers precursor-mature mappings that have different ids) and novel miRNAs with miRDeep2 quality score lower than 5 were filtered out. The generated count-file is presented in *Supplement 2*.

*Quality assessment of count-data in DESeq2*

The count-file stated in *Supplementary 2* was loaded in DESeq2 1.36.0 as a count matrix in R 4.2.0 on a MacBook Pro, with a 2.8 GHz Intel Core i7 processor and 16GB memory. The following data was used as a configuration file loaded as colData for the analyses:

**Table S3.** **Descriptive information used in association with the miRNA-data of the sampled cats for statistical analyses in DESeq2**. Samples are coded according to *Table 5* and *Table S1*. HCM=hypertrophic cardiomyopathy; DOM=domestic mixed breed cat; NFO=Norwegian Forest cat; 1-6=paired individuals; M=male; F=female; I=living indoor only; O=allowed access to outdoors.

| cat | assessment | breed | pair | age | sex | weight | house |
| --- | --- | --- | --- | --- | --- | --- | --- |
| A1 | HCM | NFO | 1 | 6.45 | F | 6.0 | O |
| A3 | HCM | DOM | 2 | 8.96 | M | 6.1 | I |
| A4 | Normal | DOM | 2 | 10.7 | M | 5.2 | O |
| B1 | Normal | NFO | 3 | 8.87 | F | 4.7 | I |
| B2 | Normal | DOM | 4 | 5.22 | M | 7.3 | O |
| B3 | HCM | DOM | 4 | 4.32 | M | 5.8 | O |
| B4 | HCM | NFO | 3 | 12.0 | F | 4.6 | I |
| C1 | Normal | DOM | 5 | 13.8 | M | 4.5 | O |
| C2 | HCM | NFO | 6 | 2.43 | M | 8.4 | O |
| C3 | HCM | DOM | 5 | 13.7 | M | 4.4 | O |
| C4 | Normal | NFO | 6 | 3.73 | M | 7.0 | I |
| D1 | Normal | NFO | 1 | 8.30 | F | 3.6 | O |

Only counts with row sums ≥10 or row means ≥5 were kept in the different datasets when size-factors were estimated. Stratification analysis was performed on shifted logarithm and regularized log transformed data and evaluations illustrated in PCA-plot, dendrogram of sample-to-sample distances (*Fig. 1* and *Fig. S3,* respectively) and heatmaps of count matrixes (*Fig. S4*). Both transformations of the count data generated similar results, hence only the results for the shifted logarithm data is presented here.

**Figure S3**. **Dendrogram/heatmap presenting sample-to-sample distances in the count-data**. Samples are coded according to *Table 5*. DOM=domestic mixed breed cat; NFO=Norwegian Forest cat; HCM=hypertrophic cardiomyopathy.

**Figure S4**. **Heatmap of miRNA-count matrix**. Samples are coded according to *Table 5*. DOM=domestic mixed breed cat; NFO=Norwegian Forest cat; F=female; M=male; HCM=hypertrophic cardiomyopathy.

*Simplified and explanatory scripts for statistical analysis in DESeq2*

#Load library

library(DESeq2)

#read in counts saved as text/csv-file by read.delim or read.csv add sep=";" or sep="\t" if relevant

cats <- read.csv("miRNA_count_table.csv", sep="\t", row.names="miRNA_identifier")

#set data as matrix

miRNA <- as.matrix(cats)

#add info for coldata

coldata <- read.csv("miRNAconfig.csv", sep=";", dec=".", row.names=1)

#check info in both matrixes

all(rownames(coldata) %in% colnames(miRNA))

all(rownames(coldata) == colnames(miRNA))

#set variables as factors

coldata$assessment <- factor(coldata$assessment)

coldata$breed <- factor(coldata$breed)

coldata$sex <- factor(coldata$sex)

coldata$pair <- factor(coldata$pair)

#set up the model

dds <- DESeqDataSetFromMatrix(countData = miRNA, colData = coldata, design = ~breed + assessment + breed:assessment)

#make sure R knows which is the reference level if alphabetical order is not ok

dds$assessment <- relevel(dds$assessment, ref = "Normal")

dds$group <- factor(paste0(dds$assessment, dds$breed))

#pre-filter low counts, manual suggest using a row sum of 10 counts or more

dds <- estimateSizeFactors(dds)

keep <- rowSums(counts(dds, normalized=TRUE)) >= 10

dds <- dds[keep,]

#differential expression analysis

dds <- DESeq(dds)

res <- results(dds)

#check results from the run, in ordered list following adjusted p-value

res <-res[order(res$padj),]

res

summary(res)

#Check result names to test with contrast

resultsNames(dds))

#Use contrast to pull comparisons between groups

resAssessment <- results(dds, contrast=c("assessment", "HCM", "Normal"))

resContrastGroup <- results(dds,contrast=c("group", "NormalDOM", "NormalNFO"))

################

##To test if a simpler model is ok

full_model <- ~ breed + assessment + breed:assessment

reduced_model <- ~ breed

#Set up likelihood ratio test

dds <- DESeqDataSetFromMatrix(countData = miRNA, colData = coldata, design = full_model)

dds <- estimateSizeFactors(dds)

keep <- rowSums(counts(dds, normalized=TRUE)) >= 10

dds <- dds[keep,]

#Run test, and in case the adjusted p-value for a miRNA pass the significance threshold (here BH-padj < 0.1) then consider use the more complex model instead.

dds_lrt <- DESeq(dds, test="LRT", reduced= reduced_model)

resModel <- results(dds_lrt)

**Validation of identified differentially abundant miRNA with qRT-PCR**

Assays for validation of differentially abundant levels of identified miRNA from the miRNome study were developed and provided by Qiagen. Prior to experimental runs of WB-samples optimisation was performed to establish appropriate volumes for total RNA extraction, input levels of total RNA for cDNA synthesis, and tests of stability of individual assays designed for the cat specific miRNA A2_1436. Standard curves for both assays are plotted in *Fig. S5* for reference.

Following establishment of Ct-values for each miRNA and sample, statistical analysis was performed to rule out effects of sample storage time. This was done by multivariate analysis in JMP (JMP Statistical Discovery LLC, Cary, NC, USA), where the normalised differentially abundant levels of each miRNA were modelled stepwise against the effects of “age of sample”, “breed”, “assessment” and “sex”, evaluating miRNAs with a p-value threshold of <0.2. Cat DOM15 was excluded from analysis, due to later identified to have had anaemia at date of sampling, hence evaluation was performed on 23 cats according to *Table S2*. Based on the results from the analysis (see *Table S4*) one more cat was excluded from the subsequent statistical analysis based on short storage time negatively influencing the dataset.

Statistical evaluation of expressed miRNA in EDTA-WB was performed in JMP based on Wilcoxon/Kruskal Wallis Tests. Some groups had too few samples to run, *e.g.* comparison between sex and assessment for NFO. The fold change difference in abundance of *miR-204-5p* between healthy and HCM-affected NFO was -0.2. The remaining comparisons are presented in *Table S5*


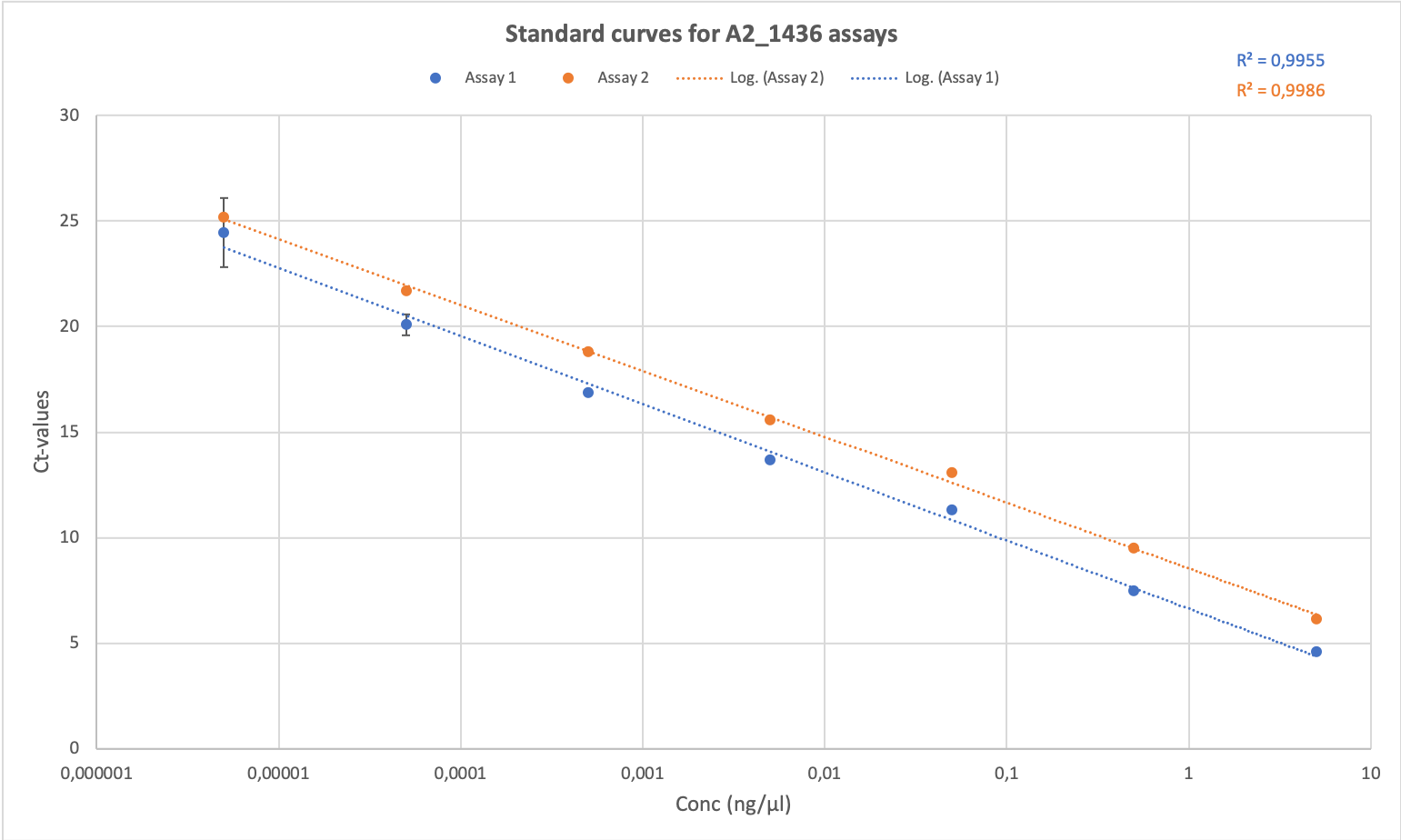


**Figure S5. Standard curves based on serial dilution of A2_1436 control for stand-alone assay**. Samples were run in duplicate and bars represents standard error for each assay and concentration.

**Table S4. Statistical significance of storage time on miRNA.** Table depict established p-values for storage time following stepwise multivariate regression analysis for a model that also included the parameters of “breed”, “sex” and “assessment”, compared with differentially abundant levels of miRNA. A p-value of <0.05 was regarded as significant and is marked with * for highlight. None of the other parameters, except breed as pointed out in manuscript, passed the significant threshold in the tested model. Abbreviations; DOM=domestic cat; NFO=Norwegian Forest cat; CHF=congestive heart failure

| *Effect of storage time* | | | | | |
| --- | --- | --- | --- | --- | --- |
| All cats (n=24) | | Exclusion of NFO1 and DOM15 (n=22) | | Exclusion of NFO1, DOM15, and cats with CHF (n=18) | |
| miRNA | **p-value** | **miRNA** | **p-value** | **miRNA** | **p-value** |
| *miR-125b-5p* | 0.6286 | *miR-125b-5p* | 0.7883 | *miR-125b-5p* | 0.5820 |
| *miR-375-3p* | 0.8066 | *miR-375-3p* | 0.8681 | *miR-375-3p* | 0.7712 |
| *let-7c-5p* | 0.1136 | *let-7c-5p* | 0.1184 | *let-7c-5p* | 0.1453 |
| *miR-144-5p* | 0.0533 | *miR-144-5p* | 0.0652 | *miR-144-5p* | 0.0712 |
| *miR-151a-3p* | 0.0190* | *miR-151a-3p* | 0.8942 | *miR-151a-3p* | 0.7753 |
| *miR-3059-5p* | 0.4844 | *miR-3059-5p* | 0.5863 | *miR-3059-5p* | 0.3722 |
| *miR-330-5p* | 0.0186* | *miR-330-5p* | 0,0783 | *miR-330-5p* | 0.0711 |
| *miR-331-3p* | 0.0418* | *miR-331-3p* | 0.0234* | *miR-331-3p* | 0.0510 |
| *miR-3613-5p* | 0.9333 | *miR-3613-5p* | 0.4525 | *miR-3613-5p* | 0.5801 |
| *miR-99a-5p* | 0.5819 | *miR-99a-5p* | 0.7089 | *miR-99a-5p* | 0.6341 |
| *miR-98-5p* | 0.1067 | *miR-98-5p* | 0.1175 | *miR-98-5p* | 0.1720 |
| *miR-26b-5p* | 0.3752 | *miR-26b-5p* | 0.2288 | *miR-26b-5p* | 0.3082 |
| *miR-326* | 0.6623 | *miR-326* | 0.8199 | *miR-326* | 0.4059 |
| *A2_1436* | 0.1186 | *A2_1436* | 0.1132 | *A2_1436* | 0.1710 |
| *miR-150-5p* | 0.7434 | *miR-150-5p* | 0.6927 | *miR-150-5p* | 0.5160 |
| *miR-146a-5p* | 0.8059 | *miR-146a-5p* | 0.6115 | *miR-146a-5p* | 0.6620 |
| *miR-204-5p* | 0.4883 | *miR-204-5p* | 0.7643 | *miR-204-5p* | 0.7729 |

**Table S5**. Statistical evaluation of significant differences of miRNAs between DOM-samples of pre-clinical cats with HCM and HCM-affected cats in CHF.

| *Effect of CHF on stated miRNA* (DOM15 excluded, n=14) | |
| --- | --- |
| miRNA | **p-value** |
| *miR-125b-5p* | 0.1198 |
| *miR-375-3p* | 0.6714 |
| *let-7c-5p* | 0.6714 |
| *miR-144-5p* | 0.4795 |
| *miR-151a-3p* | 0.3113 |
| *miR-3059-5p* | 0.1182 |
| *miR-330-5p* | 0.7773 |
| *miR-331-3p* | 1.0000 |
| *miR-3613-5p* | 0.6714 |
| *miR-99a-5p* | 0.0477* |
| *miR-98-5p* | 0.5716 |
| *miR-26b-5p* | 0.6714 |
| *miR-326* | 0.8875 |
| *A2_1436* | 0.2579 |
| *miR-150-5p* | 0.1573 |
| *miR-146a-5p* | 0.1573 |
| *miR-204-5p* | 0.3961 |

**Table S6. Fold change results from validation study of miRNA in feline WB.** Samples were based on EDTA-WB stored in -80°C for up to 16 years. Cat NFO1, DOM4, DOM7-8, DOM13 and DOM15 were excluded from the analysis. A p-value of <0.05 was regarded as significant and is marked with * for highlight. Abbreviations; HCM=hypertrophic cardiomyopathy; DOM=domestic cat; NFO=Norwegian Forest cat; FC=fold change, NA=not applicable due to too few samples.

| *Breed* | | | | | |  | |  | |  |
| --- | --- | --- | --- | --- | --- | --- | --- | --- | --- | --- |
| All cats (n=18) | | | All HCM (n=14) | | |  | |  | |  |
| miRNA | **FC** | **p-value** | **miRNA** | **FC** | **p-value** |  | |  | |  |
| *miR-125b-5p* | 1.3 | 0.477 | *miR-125b-5p* | 4.0 | 0.157 |  | |  | |  |
| *miR-375-3p* | -1.0 | 0.859 | *miR-375-3p* | -1.1 | 0.888 |  | |  | |  |
| *let-7c-5p* | 1.9 | 0.013* | *let-7c-5p* | 5.6 | 0.007* |  | |  | |  |
| *miR-144-5p* | 1.6 | 0.021* | *miR-144-5p* | 4.1 | 0.024* |  | |  | |  |
| *miR-151a-3p* | 1.9 | 0.255 | *miR-151a-3p* | 2.3 | 0.227 |  | |  | |  |
| *miR-3059-5p* | 3.5 | 0.016* | *miR-3059-5p* | 2.1 | 0.322 |  | |  | |  |
| *miR-330-5p* | 2.0 | 0.033* | *miR-330-5p* | 1.9 | 0.066* |  | |  | |  |
| *miR-331-3p* | 2.2 | 0.016* | *miR-331-3p* | 3.4 | 0.011* |  | |  | |  |
| *miR-3613-5p* | 2.1 | 0.026* | *miR-3613-5p* | 2.2 | 0.048* |  | |  | |  |
| *miR-99a-5p* | -2.1 | 0.657 | *miR-99a-5p* | 5.6 | 0.396 |  | |  | |  |
| *miR-98-5p* | 1.8 | 0.010* | *miR-98-5p* | 5.5 | 0.007* |  | |  | |  |
| *miR-26b-5p* | 1.8 | 0.016* | *miR-26b-5p* | 2.8 | 0.011* |  | |  | |  |
| *miR-326* | 1.4 | 0.183 | *miR-326* | 1.5 | 0.203 |  | |  | |  |
| *A2_1436* | 1.6 | 0.182 | *A2_1436* | 2.3 | 0.157 |  | |  | |  |
| *Sex* | | | | | | | | | | |
| All cats (n=18) | | | All NFO (n=8) | | | All DOM (n=10) | | | | |
| miRNA | **FC** | **p-value** | **miRNA** | **FC** | **p-value** | **miRNA** | **FC** | | **p-value** | |
| *miR-150-5p* | 0.8 | 0.925 | *miR-150-5p* | -0.9 | NA | *miR-150-5p* | 0.8 | | NA | |
| *miR-146a-5p* | -0.9 | 0.779 | *miR-146a-5p* | -0.9 | NA | *miR-146a-5p* | -0.9 | | NA | |
